# Adult attention-deficit/hyperactivity disorder and nicotine withdrawal: a qualitative study of patient perceptions

**DOI:** 10.1186/s12888-016-0911-9

**Published:** 2016-07-04

**Authors:** Michael Liebrenz, Carl Erik Fisher, Romilda Nellen, Anja Frei, Anne-Catherine Biechl, Nina Hiestand, Alice Huber, Anna Buadze, Dominique Eich

**Affiliations:** Department of Forensic Psychiatry, Institute of Forensic Medicine, University of Bern, Falkenplatz 16-18, Bern, 3012 Switzerland; Department of Psychiatry, New York State Psychiatric Institute, Columbia University Medical Center, 1051 Riverside Drive, New York, NY 10032 USA; Department of Psychiatry, Psychotherapy and Psychosomatics, Psychiatric Hospital, University of Zurich, Zurich, Switzerland; Institute for General Practice and Health Services Research, University of Zurich, Pestalozzistrasse 24, 8091 Zurich, Switzerland

**Keywords:** ADHD, Nicotine, Withdrawal, Subjective, Qualitative, Narrative

## Abstract

**Background:**

Nicotine use has been reported to ameliorate symptoms of Attention-Deficit/Hyperactivity Disorder (ADHD). Furthermore, adults with ADHD have a relatively high prevalence of cigarette smoking and greater difficulty abstaining from smoking. Overall, though, there is scant literature investigating the beliefs, perceptions and experiences of smokers with ADHD regarding smoking cessation and withdrawal.

**Methods:**

Our participants (*n* = 20) fulfilling criteria for ADHD and a past or current dependence from nicotine were recruited from the in- and outpatient clinic of the Zurich University Psychiatric Hospital and the Psychiatric Services Aargau (Switzerland). We conducted in-depth interviews to explore their motivations to quit, past experiences with and expectations about quitting using a purposeful sampling plan. The sample was selected to provide diversity in relation to level of nicotine dependence, participation in a smoking-cessation program, gender, age, martial status and social class. Mayring’s qualitative content analysis approach was used to evaluate findings.

**Results:**

Adult smokers with ADHD had made several attempts to quit, experienced intense withdrawal symptoms, and relapsed early and often. They also often perceived a worsening of ADHD symptoms with nicotine abstinence. We identified three motives to quit smoking: 1) health concerns, 2) the feeling of being addicted, and 3) social factors. Most participants favored a smoking cessation program specifically designed for individuals with ADHD because they thought ADHD complicated their nicotine withdrawal and that an ADHD-specific smoking cessation program should address specific symptoms of this disorder.

**Conclusions:**

Since treatment initiation and adherence associate closely with perception, we hope these findings will result in better cessation interventions for the vulnerable subgroup of smokers with ADHD.

## Background

Adults diagnosed with Attention- Deficit/Hyperactivity Disorder (ADHD) exhibit significantly higher rates of harmful use of or dependence on nicotine than persons without this neuropsychiatric disorder, with comorbidity rates as high as 35 to 55 % in United States samples [1, 2]. Similar comorbidity rates are found in Europe [3].

Patients with ADHD suffer from symptoms of inattention and hyperactivity-impulsivity that result in a diverse range of psychosocial impairments [4]. Small, prospective clinical trials have suggested that nicotine may reduce symptoms of ADHD [5, 6], supporting a “self-medication hypothesis;” i.e., some individuals with ADHD may begin smoking as an attempt to manage deficits in attention and concentration [7, 8]. The self-medication hypothesis is further supported by research on patients with ADHD showing beneficial effects of nicotine on concentration [9], sustained attention [10], and regulation of emotional dysfunction [11]. Although the self-medication hypothesis is not undisputed [12], it may help explain why patients with ADHD begin smoking at an earlier age, smoke more frequently, and are less likely to maintain abstinence compared to smokers without ADHD [13–15].

More recent research on ADHD and smoking has investigated the link between specific ADHD subtypes (e.g., combined type, predominantly inattentive type, and predominantly hyperactive-impulsive type) and certain clinical characteristics, including levels of nicotine use, withdrawal symptoms, abstinence rates, treatment readiness, and treatment response [2, 13, 15, 16]. Results from these few studies are mixed, but one study of 276 adolescents with ADHD and a comorbid (non-nicotine) substance use disorder (SUD) did not find differences in treatment response among ADHD subtypes, implying that subtype designation may have limited relevance for treatment planning [16].

In a previous study published in the same journal we explored how patients with adult ADHD, who currently smoked, viewed the relationship (or link) between nicotine use and ADHD [17]. We identified two explanatory models linking ADHD and tobacco use: *smoking as attempt of self-medication* and *smoking as a social behavior*. Participants viewed the use of nicotine rather positively—considering it a therapeutic aid—and they reported positive effects on psychopathological symptoms (relieving “inner tension”), cognitive, and social functioning. In the present study, we expand this work by focusing specifically on beliefs, perceptions and experiences of smokers with ADHD regarding smoking cessation and withdrawal.

In the general population, non-ADHD smokers attribute cigarette smoking to beneficial psychological, physiological and social effects [18, 19]; experience quitting as difficult [20]; and frequently relapse [21, 22]. With this in mind, we aimed to assess the motivations, expectations, and experiences of patients with ADHD with regard to smoking cessation and withdrawal, since previous studies have demonstrated that these subjective factors at least partially predict future quitting attempts [23] and relapse [24]. We also explored patients’ reasons for participating in a cessation program and on their thoughts about an “optimal” smoking cessation intervention.

Especially in light of the lack of evidence-based guidelines for the treatment of comorbid ADHD and SUD [25], these questions are of clinical importance. It is increasingly understood that treatment plans that do not take into account subjective perceptions are unlikely to increase help-seeking behavior and are therefore associated with negative treatment outcomes [26].

## Methods

### Participants and procedure

Data were collected during a series of qualitative interviews with adult smokers with ADHD in treatment either at the ADHD consultation service at the Centre for Addiction Disorders, an outpatient facility of the Zurich University Hospital (12) or the Psychiatric Services Canton Aargau (8).

A mixed method of purposeful sampling and saturation sampling principles was used. The sample was selected to provide diversity in relation to (1) level of nicotine dependence (very low to very high); (2) clinical experience (previous in- and outpatient treatment episodes), including comorbidity (affective disorders, neurotic disorders, personality disorders, gender (male/female), age (25–54), martial status (married, single, divorced), social class (professional, skilled, unskilled, unemployed, recipient of welfare or disability compensation) and immigration status (citizens, permanent residents). We also sampled for participants who had participated in the smoking cessation program (8) and for those who had not (12).

Zurich participants were recruited from a larger sample of 134 participants who had participated in an epidemiological study of the association between ADHD and tobacco use [3, 27]. Depending on the questionnaire used, participants reached average test scores of 40.0 (SD = 11.13) on WURS-k, 29.2 (SD = 8.99) on ADHS-SB and 15.4 (SD = 7.7) on the newly developed SCL-ADHD scale [28]. In this sample, fifty-five participants of the larger epidemiological study qualified for inclusion: they were diagnosed with ADHD, currently using tobacco, and at least 18 years of age. We were able to reach 48 of them and 12 agreed to participate.

Prior being interviewed, eight out of these 12 subjects recruited had participated in a smoking cessation program that was modeled after recommendations by Cornuz et al. [29] and the U.S. Public Health Service, Office of the Surgeon General [30]. It consisted of five standardized counseling interviews based on cognitive behavioral therapy techniques. Additional pharmacological support, including bupropion and nicotine replacement therapy, was offered to participants. The counseling sessions took place within the first three months, and two additional follow-up visits were scheduled at six and 12 months. Additional appointments were made at the request of the participants. Initial counseling focused on smoking history, psychoeducation about nicotine dependence, trigger situations, motivations for smoking, pharmacotherapy, and developing a plan to quit. Thereafter, participants were counseled on emotional reactions to success or failure, withdrawal symptoms, weight gain, and behavioral strategies to prevent relapse. During all sessions participants received strong encouragement from the therapist.

Additionally, we interviewed eight subjects from the Psychiatric Services Aargau who had not participated in a cessation program to obtain a greater variation of themes and motives.

Subjects who declined specifically named barriers to participation in a few instances. Lack of time was the most common reason why potential participants declined inclusion in this study. Additionally, three potential participants agreed to be interviewed, but missed their appointment. Attempts to contact these subjects again were not successful.

Interviews explored the following topics: 1) past experiences with smoking cessation and withdrawal, including strategies used; 2) motivations, expectations, and reasons for participating in a cessation program; 3) thoughts about an “optimal” smoking cessation intervention; and 4) perceptions about a smoking cessation program specifically designed for adults with ADHD.

### Assessment of ADHD and nicotine dependence

Prior to inclusion, all participants had a complete evaluation of their ADHD in accordance with current diagnostic recommendations [31] using the Wender-Reimherr Interview (WRI) a German version of the American Wender-Reimherr Adult Attention Deficits Disorders Scale (WRAADDS), the Wender Utah Rating Scale (WURS-k) [32], and the Attention-Deficit/Hyperactivity Self-Report Scale (ADHS-SB) [33].

Additionally, researchers had access to a complete clinical chart on each subject, including biographical data and comorbid psychiatric diagnosis according to the 10th revision of the International Statistical Classification of Diseases and Related Health Problems (ICD-10). Nicotine dependence was assessed using the six-item Fagerstrom Test for Nicotine Dependence (FTND) [34], and abstinence (for those participating in the smoking cessation program) was evaluated by self-report and expired-air carbon monoxide level [35].

### Data collection and analyses

The qualitative interviews each lasted between 20 to 60 min and were digitally recorded and transcribed *verbatim*. Interviews were conducted by two researchers (AF/RN), and participants chose the interview locations so they felt free to fully express their own views and perceptions [36]. Interviews began with narrative opening questions, and a topic guide was used as part of a flexible interview framework in order to capture beliefs that were not spontaneously addressed by participants’ initial narratives.

Mayring’s qualitative content analysis approach was used to evaluate findings. This framework constitutes an controlled approach for empirical and methodological qualitative analysis of texts within their context of communication, following content analytical rules and step-by-step models, without rash quantification and not aiming to test a specific hypothesis [37]. In other words, the data were allowed to “speak for themselves”, as opposed to approaching the data with existing presumptions. Interview data were coded using an inductive qualitative procedure. The resulting categories were discussed by the research team to validate ratings and achieve consensus. AF and RN applied the final code, and consistency was confirmed through blind dual coding of transcripts with ML. Participant recruitment continued until we reached saturation of the data, in that there were no new themes emerging and we had tested all the categories for disconfirming variations. MAXqda software [38] was used for text management and interpretation. This study was authorized by the local ethics committee.

We reported this study using COREQ guidelines [39].

## Results

A total of 20 participants demonstrating a range of nicotine dependence were interviewed. The mean age of the participants was 38.95 years ± SD 9.55 (median 42.0 years, range 25 to 54 years). At the time of interview, participants smoked an average of 24.35 ± SD 9.64 cigarettes per day (median 26.50, range 1 to 35). All subjects were receiving psychotropic medications, most commonly stimulants, but also antidepressants and (in two cases) antipsychotics. Eight (40 %) participants used other substances, most commonly alcohol, while the other twelve (60 %) abstained from any substance.

Participants’ characteristics, diagnosis, and tobacco consumption patterns are described in Table 1.

**Table 1:** Participants’ characteristics, diagnosis, and nicotine consumption patterns

		*n*	%
Number of participants		20	
Gender			
	female	10	(50.0)
	male	10	(50.0)
ADHD subtype according to DSM-IV TR			
	combined	13	(65.0)
	hyperactive/impulsive	6	(30.0)
	inattentive	1	(5.0)
Nicotine dependence according to Fagerström			
	very low	2	(10.0)
	low	1	(5.0)
	moderate	1	(5.0)
	high	8	(40.0)
	very high	8	(40.0)
Medication at time of interview			
	stimulants	13	(65.0)
	antidepressants	11	(55.0)
	mood stabilizers	2	(10.0)
	antipsychotics	2	(10.0)
Previous phases of nicotine abstinence^a^			
	none	5	(25.0)
	<1 month	5	(25.0)
	1 - 12 months	6	(30.0)
	>1 year	4	(20.0)
Number of comorbid psychiatric diagnosis groups except for ADHD and nicotine dependence			
	none	2	(10.0)
	one	10	(50.0)
	more than one	8	(40.0)
Comorbid psychiatric diagnosis groups except substance use disorders (ICD-10)			
	schizophrenia	1	(2.4)
	mood disorders	3	(15.0)
	neurotic disorders	4	(20.0)
	personality disorders	3	(15.0)

^a^as of the time of interview, self reported, where possible chart verified

### Past experiences with smoking cessation and withdrawal

Most participants, regardless of whether they had participated in the smoking cessation program, had attempted to quit smoking several times in the past. Only three subjects had never tried to stop smoking.

*“…There were certainly eight attempts [of quitting smoking]…”*

*Mrs. F.*

*“Around four – to five times…”*

*Mrs. G.*

Cessation efforts had resulted in a diverse length of abstinence ranging from days to years.

*“I stopped once for 6 months, that is a long time ago when I was 22. The last time I made it for four days.”*

*Mrs. J.*

*“…the only longer period…where I had the feeling of being out of the woods…was for one month…”*

*Mr. K.*

*“…The longest period was one year of not smoking…”*

*Mrs. G.*

Some participants considered smoking cessation relatively easy, with only minor complications:

*“…nothing big…well, the first three days more psychologically, that I was thinking about it. But physically I did not notice anything…certainly more nervous though”*

*Mrs. H.*

*“…I experienced no withdrawal symptoms, maybe a little restlessness…”*

*Mrs. E.*

The majority of participants, however, experienced attempts to quit as highly stressful and described severe difficulties during those attempts, regardless of whether they had made a conscious decision to quit or they were forced to quit by external circumstances.

*“…and stopping is so tedious, it fills your mind all day and all night, that you are always thinking about it…you always think you must not smoke, it becomes the primary objective. And then I get in to panic that will go on until life’s end…it is exhausting…”*

*Mrs. J.*

*“I noticed then, that it needs, after such a long time, I am now 53 years old, and I got used to a lot of this stuff and then I noticed the motivation is there, but it is really tough to pull it through…”*

*Mr. A.*

Most often, subjects had experienced symptoms of irritability, nervousness, and restlessness, sometimes accompanied by difficulty sleeping or symptoms of depression and anxiety.

*“Nervousness, restlessness, the permanent back and forth, not knowing what it is all about [starts laughing]…I forgot to go get groceries, and I just noticed it at night, and then I forgot it again, because I was so busy, and then I had to get out again…back then…I started sweating and it was difficult to concentrate, but no sleeplessness…”*

*Mrs. F.*

*“…I started to cry because of little things, although I am usually not someone who starts crying, that was bad, tragic…and the irritability, I could not bear anything, I had to cry the entire time and was furious…”*

*Mrs. G.*

*“…one time I had sleep issues, the other times I did not have that. And then the devil in your body. That was the worst for me. Always: now a cigarette, a cigarette, that would be good now, simply, such a chase, now you have to have one…”*

*Mrs. G.*

*“…the first few days were nasty…it was a physical malaise and…also psychologically. I am not easy to talk to, on the edge and bad tempered…”*

*Mr. K.*

Some participants who experienced these difficulties described the use of other psychotropic substances to cope:

*“…I noticed that I got depressed and aggressive and that something was missing…and than I started to drink more, and that was a direct consequence. That is why I knew it was alright to drink a bit more…”*

*Mr. A.*

Others noticed a change in their caffeine use or eating:

*“ …for a time I drank more coffee…”*

*Mrs. E.*

*“…I had a much increased appetite for candy and sweets, what I usually don’t have…”*

*Mr. K.*

Less frequently, other side effects were noted; for example, a perceived increase in weight:

*“… I was totally shocked when I stopped [without counseling], what happened back then…when I gain weight that is a huge problem. I can not accept that, I don't want to be 4–5 kg more than I am now, and I don't want to be a nervous wreck. I can not afford that with my children. The last time I tried to quit I almost had a nervous breakdown, and that was shocking.”*

*Mrs. G.*

Notably, almost all participants who experienced difficulties associated these symptoms with immediate nicotine withdrawal. Since symptoms of ADHD can be difficult to differentiate from nicotine withdrawal symptoms, participants where further asked to elaborate on this issue. The question of whether smoking cessation had a lasting influence on symptoms of ADHD was addressed by half of the participants. While one participant denied that symptoms of ADHD had gotten worse, all others were convinced that smoking cessation had resulted in a worsening of ADHD symptoms even after immediate physical symptoms of withdrawal had subdued.

*“… I get even more erratic and aimless, I walk the entire house and don’t remember what I had planned to do, why and where, I just jump around…”*

*Mrs. J.*

*“… It amplified [symptoms]…my strategies to calm myself down were not working anymore…”*

*Mr. I.*

Independent of withdrawal symptoms, some participants viewed external social factors as the most problematic experience after ceasing the use of cigarettes.

*“Actually I didn’t have big problems, during that time, I mean the biggest problem I had was my partner, because he smokes and we were together for such a long time, and then I was smoking with him and that was a problem…”*

*Mrs. C.*

Only a minority of participants had tried to quit smoking without treatment, conventional or otherwise. Subjects had used medications (bupropion, nicotine replacement therapy) as well as alternative methods like acupuncture (two) and magentopathy (one) in various combinations. While pharmaceutical support was experienced as somewhat helpful in attenuating symptoms of withdrawal, acupuncture was not considered helpful and “magnetopathy” was thought to have a positive but transient influence:

*“…what helped me was Zyban® and sports…”*

*Mrs. G.*

*“… before that I tried acupuncture, twice with permanent needles, once just with regular needles…did not help…”*

*Mrs. F.*

*“I was working with a magneopath and I was smoking 24 cigarettes a day back then, and then one week only two cigarettes, that was a good experience…”*

*Mrs. D.*

Many subjects emphasized that in previous attempts to cease smoking an intensive physical exercise program was very helpful.

*“…I did do a lot of sports, but at the end of the day, I did not find it difficult…”*

*Mrs. C.*

Other measures included the use of licorice for oral stimulation and reading Allen Carr’s *Easy Way to Stop Smoking* [40] in order to enhance motivation.

*“I had a phase four to five years ago and that went relatively well…relatively soon smoking was not an issue anymore, actually the opposite, I found it disgusting. The missing stimulation was difficult, I started with licorice as a replacement, because I was missing the sucking, so I began to suck on that licorice [laughing], that helped, but I permanently had a piece of wood in my mouth, but it also calmed me…”*

*Mrs. E.*

*“I had a time when I was not smoking for 15 months… I read a book by Alan Carr, that really helped me and I sill believe if I had the money that I would like to take one of those courses.*

*Mrs. C.*

### Motivations, expectations, and reasons for participating in a cessation program

All subjects addressed the issue of motivation, often spontaneously as part of their initial narrative, regardless of whether they had decided to participate in the cessation program. We identified three themes regarding motivation, but there was significant overlap between themes, and eight participants described more than one motivational factor.

The most commonly expressed theme was *health concerns.* Subjects were afraid of serious negative consequences to their personal wellbeing from smoking. Additionally, many subjects described perceived negative effects on their physical resilience.

*“…from my point of view the damage (of smoking) is mostly physical in nature, it puts a limitation on your life, on your fitness, and in the long term all these damages to your health, yes…”*

*Mr. B.*

*“…now it is really, the exercise, the new kinesthesia which you get when you say no, I don’t want to give up now, because that just does not fit together, I cannot work out 5 hours per week and then smoke 10 cigarettes per day, that does not work and this is where I draw the line…”*

*Mr. B.*

*“It all starts with your health, that it is somehow harmful, and that you also feel it, I mean I was not smoking for one year and I know how it is not to be smoking, how you feel with that…”*

*Mrs. G.*

An equally important motivational factor for many individuals was the *feeling of being addicted,* a limitation they described as a lack of personal freedom.

*“[disadvantages] are for me more of psychological nature, rather than physical, because I go to the doctor on a regular basis to get a check up, I also get lung X-rays frequently and then I am always relieved that I can continue to smoke. It is more the lack of freedom… that I am dependent on these cigarettes…”*

*Mrs. D.*

*“…and the really bad thing is this addiction, I find this worse than all the damages to health. Just because you must have one, it is not your will that is deciding, now I am gonna have a smoke and now not, it is not like eating chocolate, you really must [smoke] …”*

*Mrs. C.*

As expected, *social factors* were also frequently mentioned. Immediate family members, relatives and significant others were important external motivational influences. Many participants felt a moral obligation to avoid harming their offspring. Subjects of both genders described previous phases of abstinence during pregnancy and caring for children.

*“… I mean when I first stopped smoking my motivation was my family, my two kids, my two babies at home, that was enough back then…”*

*Mr. B.*

*“…[I stopped smoking] during pregnancy and lactation…it was clear to me that I would have had remorse…”*

*Mrs. H.*

*“During the last pregnancy… I stopped smoking for two to three weeks…I made sure that I did not inhale…”*

*Mrs. F.*

Interestingly, two subjects specifically viewed themselves as socially excluded or even harassed by non-smokers and felt that these feelings had contributed to their decision to quit smoking.

*“Today it is also partially difficult, where can you go, without getting looked at…”*

*Mrs. F.*

Less surprisingly, financial stress was another frequently mentioned external social factor.

*“I don’t see a benefit, you know every reasonable human sees first, that it costs a lot of money, which you could use otherwise…”*

*Mrs. J.*

*“…and also the cost, that is something…”*

*Mrs. F.*

### Thoughts about an “optimal” smoking cessation intervention

Our study participants offered their own suggestions for the design of smoking cessation programs. Interestingly, many subjects viewed an inpatient treatment setting in a “specialized” hospital as desirable and were ready to participate in such treatment even for several weeks. They associated inpatient treatment with less severe withdrawal symptoms because of continuous access to health care professionals and protection against relapse.

*“maybe something more like an inpatient program, a little bit like a vacation program, with occupational therapy, and when I have a crisis, someone is right there for me. I have been thinking about entering a clinic…for a smoking cessation program, and then I think this is a bit too much [for smoking], everybody else can do without it, but maybe it would be something for me… if there would be care 24/7.”*

*Mrs. D.*

*“and what I would immediately do is to go somewhere for two or three weeks like a heroin addict. I think it is kind of sad, that something like this does not exist for smokers, because I have seen this with my brother who is a heroin addict. I don't see a difference between us [smokers] and heroin-addicted humans. In my perception I am chasing after a cigarette just as he is chasing after an injection, just another form, same thing. It’s a kind of pity that there is no place where I can have a controlled withdrawal…”*

*Mrs. G.*

Other subjects suggested even more extreme methods, ranging from a “sleep regimen” to being abandoned on a distant island. Furthermore, several participants were interested in hypnosis.

*“… I believe something would have to be changed inside my head [laughs]. I do have the feeling that, I don’t know if it is possible, to participate in a week long “sleeping regimen” in a hospital…*

*Mrs. J.*

*“Or something without side effects, that doesn’t bother and doesn't stress me…and I have to add, that I have something in my head that I would like to try, which has fascinated me for a long time…hypnosis…”*

*Mrs. G.*

There was some overlap in this theme with the desire for a “quick fix” or miracle cure, something fast, with no symptoms of withdrawal.

*“…the best idea would be to find the switch in your brain, where you can just turn it off…”*

*Mrs. F.*

*“…and of course you wish that someone just has a solution for you…”*

*Mrs. E.*

*“I haven’t given up on it yet, I really want to quit, but, it is still incomprehensible for me, how I am supposed to do that, how I can outsmart myself. Last time it just did not happen, I am still waiting, it sounds stupid, for an inspiration, for something that I suddenly can manage it with.”*

*Mrs. F.*

Some participants suggested that a group setting might be more beneficial than individual counseling.

*“…I believe that if it is done in a group of other persons who are affected, that say we all want to stop now, and are motivating each other or can talk about difficulties, than it might create good feelings, that one is not alone with this.”*

*Mr. K.*

Interestingly some subjects felt that financial penalties or a vivid description of the harmful consequences of tobacco use might lead to success.

*“…Education, ah, ‘shock therapy,’ although some say it is not helpful, but it supported me a lot, back when a friend of mine was diagnosed with lung cancer and my family practitioner told me that I have chronic ‘smokers cough’ and what that all means and what kind of lung diseases there are, that really alarmed me… you tend to block these things out of your mind…”*

*Mrs. C.*

*“…Coaching could be more intensive and more demanding. I think there should be more pressure from the side of the therapist…and maybe even some financial commitment, defining targets and stages, and also rewards and penalties…”*

*Mr. B.*

One participant, who was aware of his dual-diagnosis, explained that he viewed psychiatric treatment for his ADHD as the primary strategy to stop smoking.

*“…In my case it would need psychiatric support, it can be done together with the treatment of ADHD, because it fits there. Because there are some studies that say nicotine helps people with ADHD, and because of that it is another hazard, and it is also an extra step for people with ADHD, because it is not just an addiction, but almost medicine.”*

*Mr. L.*

### Perceptions about a smoking cessation program specifically designed for adults with ADHD

While four subjects were reluctant to comment, one participant opposed a program designed for adults with ADHD. Another subject similarly noted that there were many smokers without ADHD who had difficulty quitting.

*“…I am not sure about it, because there are many people out there, who more or less have it, and who have lived with it without knowing…and that is why…”*

*Mrs. G.*

*“…I could not say, but I see in everybody how difficult it is [to stop smoking].”*

*Mrs. H.*

The other study subjects supported specific counseling for individuals with ADHD, as they felt that people with ADHD have unique smoking patterns. They felt that a specifically designed program should expect a worsening of ADHD symptoms during nicotine withdrawal, help develop coping strategies for these symptoms, consider inattentive symptoms and symptoms of “inner tension,” deliver support during “failures,” engage participants more actively, and involve family members.

*“…the major difference I see is that people with ADHD have a harder time quitting smoking than people without ADHD…and with me I am just scared to change, the inhibition threshold increases because you are chaotic and [when you stop] I am afraid that the chaos just increases, and that is why you wait for a more quiet moment in life…and I think these are the kind of things that people with no ADHD would not think.”*

*Mrs. C.*

*“…the form of consultation [for smokers with ADHD] should be fundamentally different. That you really look if someone can keep the attention focused on the course, that you also support more in case of failure, and that you keep at it, and keep at it, because for smokers with ADHD it is not necessarily a motivational problem… And the topic of compensation is more important, because of the strong inner tension that most experience, that is something that contributes to smoking…”*

*Mrs. E.*

*“I could imagine that it is more important to involve family and friends in smokers with ADHD, compared to other participants, because this way there would be a bit more control, responsibility, support through children, whatever…I think the social community is more important in patients with ADHD than in others…”*

*Mr. B.*

One participant suggested that screening for ADHD in smokers should be done more routinely, pointing out that he was diagnosed only at a later age.

*“…ADHD people are usually more nervous, and they are always sped up, and so on, and the most people with ADHD I know smoke, and smoke a lot, and you have to specifically reach out to these people and also the ones who do not know that they might suffer from that illness, because many people with ADHD that I know, they don’t know that they have it, so it starts earlier on…I only learned about my diagnosis when I was 50 years old…”*

*Mr. A.*

Another subject was convinced that a cessation program should not focus on the use of tobacco, but on substance misuse in general.

*“… I have a lot of experience with ADHD…someone who does not have this sort of experience, who has ADHD and has to fight with addiction, they might need more alternatives… what are these alternatives, and you have to look how it really is with ADHD, and with this inner tension, because you will definitely need something. It should not just be about smoking. The program should be about addiction. I know different people with ADHD and they all have their problems and different strategies to approach them. So we need to know about alternatives, and many individuals with ADHD really have to fight addiction and dependence. Many smoke cannabis like crazy to calm down, others use cocaine…”*

*Mr. A.*

## Discussion

This study describes the views and experiences of adults with ADHD regarding smoking cessation and withdrawal, their expectations and motivations for participation in a smoking cessation program, and their ideas about optimal support during the withdrawal process.

The vast majority of study participants had a long history of tobacco use. Sixteen subjects were rated “highly” or “very highly” dependent on nicotine according to the FTND, and many reported several previous attempts to quit smoking. Subjects had frequently relapsed after short periods of abstinence, and they described symptoms of irritability, nervousness, restlessness, insomnia, depression, anxiety, and weight gain during withdrawal. These self-reported symptoms do not significantly differ from previously reported self- and observer-rated withdrawal symptoms of self-quitters in non-clinical samples [41, 42].

Some data indicate that adult smokers with ADHD experience worse withdrawal symptoms, depressive mood swings, somatic symptoms, and craving after stopping nicotine use, compared to smokers without ADHD [43–45]. For example, McClernon et al investigated forty moderate- to heavy smoking adults with and without ADHD during a 12-day quit study; they reported greater withdrawal severity among smokers with ADHD, especially in the first 5 days, which was independent of effects on ADHD symptoms [44]. Our participants’ experiences are in accord with these findings. Fewer studies have evaluated the link between smoking cessation and potential worsening of ADHD symptoms. Some authors argue that there is a significant overlap between nicotine withdrawal symptoms and symptoms of ADHD and therefore urge caution on this subject [46]. Others, however, have reported that smoking withdrawal symptoms occur independently from changes in ADHD symptoms [44]. Our participants almost unanimously believed that smoking cessation resulted in a worsening of their ADHD symptoms, even after initial nicotine withdrawal symptoms had subdued.

Our study subjects described a wide variety of smoking intervention and self-help strategies that they had used in previous cessation attempts, including both evidence- and non-evidence-based approaches. Several had tried acupuncture, “magnetophathy,” and homeopathy. Similarly, a recent population-based study of 988 Swiss smokers found a high preference for non-evidence-based treatment strategies, including acupuncture and hypnosis, although both treatments are in few instances prescribed by physicians [47]. The authors of one previously mentioned study argue that a “lack of knowledge of their ineffectiveness, the fact that more educated subjects tend to complement effective treatments with alternative methods and the media coverage by the lay press” may contribute to the popularity of alternative treatments [47].

We identified three interrelated motives to quit smoking, though there was some overlap between motives and the majority of our participants gave multifactorial explanations. The first motive, *health concerns,* was named by the majority of the participants and was expressed most frequently. Subjects were afraid of serious future deteriorations in their health status and of negative health consequences in general. The second motive, the *feeling of being addicted*, was associated with a lack of personal freedom and independence. The third motive, *social factors*, was mentioned as frequently as the second motive, and it involved themes such as family ties, perceived social undesirability, and financial considerations. These findings are in accord with the extensive literature on factors associated with the desire to quit smoking in smokers without comorbid ADHD [48–52]. For example, Riedel et al investigated a sample of 120 adolescent smokers, and they reported that concerns about future health, current health, and physical appearance; the cost of cigarettes; and athletic performance were the most important motivators to quit smoking [50]. A recent study on “triggers” to quit smoking came to the conclusion that individual factors, such as concerns for personal health, are a culturally independent motivator to stop smoking and are more important than interpersonal factors such as societal disapproval [53].

As demonstrated by their views about an optimal smoking cessation program, participants had some misconceptions about nicotine dependence. For example, subjects did not understand its status as a substance use disorder; some thought an optimal treatment program would allow them to immediately and permanently abstain from smoking without experiencing any symptoms of withdrawal. The motive of a *desire for a “quick fix”* was common, similar to a desire for finding a “switch in the brain” or a “miracle solution.” Subjects who voiced this motive also suggested a wide variety of measures, including intensive inpatient treatment, financial penalties for non-compliance, non-evidence-based treatment strategies (e.g., hypnosis or sleep therapy), or even the fantasy of abandonment in a distant location.

Other findings in this context were more heterogeneous. Some participants thought group therapy should be part of an optimal treatment program, and another participant suggested that focusing primarily on treating ADHD would best change smoking patterns. Furthermore, none of our study subjects spontaneously expressed interest in a long-term nicotine maintenance approach, instead focusing on complete abstinence, even though by the end of the smoking cessation program no subjects were able to maintain abstinence, and half of the participants had returned to their initial pattern of tobacco use. This finding is consistent with previous reports that smokers prefer cessation to nicotine maintenance, and that some smokers even perceive nicotine replacement as harmful [54, 55].

Finally we explored perceptions about a smoking cessation program specifically designed for adults with ADHD. The majority of subjects felt a need for this type of specific counseling, as they considered their ADHD symptoms a significant complication during nicotine withdrawal. Most commonly, subjects called for a treatment protocol that would take into account worsened ADHD symptoms and prepare them with coping strategies specific for these symptoms.

This study has certain limitations: AF, a clinical psychologist, who also provided counseling as part of the smoking cessation program, conducted all interviews with SCP participants. This may have influenced participant responses on SCP. However, we also included twelve subjects from different locations, who had not participated in the SCP, broadening the sample of participant views. Furthermore data on nicotine consumption patterns are self-reported; relying on an active reconstruction process called recall, and may have resulted in a recall bias.

## Conclusions

In summary, adults smokers with ADHD had made several attempts to quit (often using non-evidence-based treatments), experienced those efforts as highly stressful, felt intense symptoms of withdrawal, and relapsed early and frequently. Subjects also frequently noted a perceived worsening of ADHD symptoms with phases of prolonged nicotine abstinence. Their motives to quit smoking were very similar to smokers without ADHD, including health concerns, the feeling of being addicted, and social factors. The majority of subjects who participated in the smoking cessation program viewed it as helpful, and they highlighted the benefits of both structure and counseling. Additionally, many participants in this study favored a smoking cessation program specifically for individuals with ADHD. Many believed that nicotine withdrawal was complicated by symptoms of ADHD and that an ADHD-specific smoking cessation program should address specific symptoms of this disorder. Ultimately, considering that both the initiation of psychiatric services and adherence to those services are closely associated with personal attitudes and beliefs, we offer these findings in the hope that future cessation interventions for smokers with ADHD might better serve this vulnerable subgroup [56, 57].

## Abbreviations

ADHD, Attention-Deficit/Hyperactivity Disorder; ADHS-SB, Attention-Deficit/Hyperactivity Self-Report Scale; FTND, Fagerstrom Test for Nicotine Dependence; ICD-10, 10th revision of the International Statistical Classification of Diseases and Related Health Problems; SUD, substance use disorder; WRAADDS, Wender-Reimherr Adult Attention Deficits Disorders Scale; WRI, Wender-Reimherr Interview; WURS-k, Wender Utah Rating Scale
